# Spatial clustering and local risk of leprosy in São Paulo, Brazil

**DOI:** 10.1371/journal.pntd.0005381

**Published:** 2017-02-27

**Authors:** Antônio Carlos Vieira Ramos, Mellina Yamamura, Luiz Henrique Arroyo, Marcela Paschoal Popolin, Francisco Chiaravalloti Neto, Pedro Fredemir Palha, Severina Alice da Costa Uchoa, Flávia Meneguetti Pieri, Ione Carvalho Pinto, Regina Célia Fiorati, Ana Angélica Rêgo de Queiroz, Aylana de Souza Belchior, Danielle Talita dos Santos, Maria Concebida da Cunha Garcia, Juliane de Almeida Crispim, Luana Seles Alves, Thaís Zamboni Berra, Ricardo Alexandre Arcêncio

**Affiliations:** 1 Graduate Program in Public Health Nursing, University of São Paulo at Ribeirão Preto College of Nursing, Ribeirão Preto, São Paulo, Brazil; 2 Graduate Program Interunit Doctoral Program in Nursing, University of São Paulo at Ribeirão Preto College of Nursing, Ribeirão Preto, São Paulo, Brazil; 3 Department of Epidemiology, School of Public Health of the University of São Paulo, São Paulo, São Paulo, Brazil; 4 Maternal-Infant Nursing and Public Health Department, University of São Paulo at Ribeirão Preto College of Nursing, Ribeirão Preto, São Paulo, Brazil; 5 Department of Public Health, Federal University of Rio Grande do Norte, Natal, Rio Grande do Norte, Brazil; 6 Department of Nursing, Londrina State University, Londrina, Paraná, Brazil; 7 Department of Neurosciences and Behavioral Sciences, Ribeirão Preto Medical School of the University of São Paulo, Ribeirão Preto, São Paulo, Brazil; Fondation Raoul Follereau, FRANCE

## Abstract

**Background:**

Although the detection rate is decreasing, the proportion of new cases with WHO grade 2 disability (G2D) is increasing, creating concern among policy makers and the Brazilian government. This study aimed to identify spatial clustering of leprosy and classify high-risk areas in a major leprosy cluster using the SatScan method.

**Methods:**

Data were obtained including all leprosy cases diagnosed between January 2006 and December 2013. In addition to the clinical variable, information was also gathered regarding the G2D of the patient at diagnosis and after treatment. The Scan Spatial statistic test, developed by Kulldorff e Nagarwalla, was used to identify spatial clustering and to measure the local risk (Relative Risk—RR) of leprosy. Maps considering these risks and their confidence intervals were constructed.

**Results:**

A total of 434 cases were identified, including 188 (43.31%) borderline leprosy and 101 (23.28%) lepromatous leprosy cases. There was a predominance of males, with ages ranging from 15 to 59 years, and 51 patients (11.75%) presented G2D. Two significant spatial clusters and three significant spatial-temporal clusters were also observed. The main spatial cluster (p = 0.000) contained 90 census tracts, a population of approximately 58,438 inhabitants, detection rate of 22.6 cases per 100,000 people and RR of approximately 3.41 (95%CI = 2.721–4.267). Regarding the spatial-temporal clusters, two clusters were observed, with RR ranging between 24.35 (95%CI = 11.133–52.984) and 15.24 (95%CI = 10.114–22.919).

**Conclusion:**

These findings could contribute to improvements in policies and programming, aiming for the eradication of leprosy in Brazil. The Spatial Scan statistic test was found to be an interesting resource for health managers and healthcare professionals to map the vulnerability of areas in terms of leprosy transmission risk and areas of underreporting.

## Author summary

Brazil has still not achieved the goal of leprosy elimination established by the World Health Organization. The diagnosis and treatment of leprosy are available and the country is striving to fully integrate leprosy services into the existing general health services. Access to information, diagnosis and treatment with multidrug therapy (MDT) remain key elements in the strategy to eliminate the disease as a public health problem, defined as reaching a prevalence of less than 1 leprosy case per 10,000 inhabitants. Thus, this study aimed to identify spatial clustering of leprosy and to classify high-risk areas in a major leprosy cluster. A total of 434 cases were identified, with 188 (43.31%) being of borderline leprosy and 101 (23.28%) lepromatous leprosy. There was a predominance of males, with ages ranging from 15 to 59 years, and 51 patients (11.75%) presented G2D. Two significant spatial clusters and three significant spatial-temporal clusters were also observed. These results can assist health services and policy makers to improve the health conditions of the Brazilian population, advancing towards the goal of elimination in Brazil.

## Introduction

Leprosy is a chronic infectious-contagious disease caused by *Mycobacterium Leprae*, an obligate intracellular bacillus that affects the skin and the peripheral nervous system [1]. Leprosy is characterized as a slowly advancing disease and, due to the characteristics of the bacillus, presents high infectiousness and low pathogenicity [1], being a potentially disabling and stigmatizing disease. For diagnosis and definition of the treatment regime with polychemotherapy (PCT), two operational classifications are used that are based on the number of cutaneous lesions, according to the following criterion: Paucibacillary Cases (PB) with up to five skin lesions; Multibacillary Cases (MB) with more than five skin lesions [2].

The disease is part of the group of neglected diseases and is relevant for public health due to its magnitude and range, causing disabilities in low-income and economically active populations [3]. Among the infectious diseases, it causes the greatest number of permanent disabilities.

The high burden of the disease signals the maintenance of the epidemiological chain of transmission, representing one of the most important epidemiological indicators [3]. Although approximately 30 years have passed since the introduction of multidrug therapy (dapsone, rifampicin and clofazimine), prevalence and incidence rates remain considerable in the country. This demonstrates that other factors play a decisive role in its causal network, such as the biology of the etiological agent, the genetic or immunological characteristics of the host, and social and economic factors, such as precarious living conditions, migration, malnutrition and poverty, among others [4,5]_._

In 2014, 31,000 cases were reported in Brazil, with a detection rate of 15.31 cases per 100,000 inhabitants and a prevalence rate of 1.56 cases per 10,000 inhabitants, putting the country second in the global ranking, only behind India [6]. Also in 2014, Brazil presented a detection rate of cases with grade 2 disability of one case per 100,000 inhabitants [7].

It should be highlighted that Brazil is the only country in the Americas that has been unable to eliminate leprosy (prevalence < 1 case per 10,000 inhabitants).

Since 2000, a movement has been ongoing in Brazil to reduce the burden of leprosy through strategies that are intended to expand the actions to the entire Health Care Network. These include early diagnosis and qualification of patient care, promoting the decentralizion of diagnosis, treatment and prevention actions to Primary Health Care (PHC), the reorganization of services, disclosure regarding the characteristics, signs and symptoms of the disease and universal access [8].

Another strong point is the identification of the most problematic areas of the disease which, once identified, can be the target or focus of healthcare or intersectorial actions, considering the relationship with social determinants [5].

In a literature review that used the descriptors ‘spatial analysis’ AND ‘leprosy’, a large number of articles related to the theme were retrieved, considering different branches or approaches to the clinical aspects of the disease [9], the evolution of the treatment (cure or abandonment) [10], disabilities and late diagnosis [11]. In general, the majority of studies aimed to provide a more exploratory description of leprosy in space, without considering the risk certain communities are exposed to in relation to others. Depending on the risk, emergency measures need to be adopted immediately to solve it or avoid the dissemination of the disease. Inferences regarding risk are an important tool for management, because they permit targets to be outlined and priority levels to be defined. Risk is traditionally focused on the quantification of the probability of negative consequences of one or more factors identified as harmful to health [12].

In view of the importance of advancing health policies in Brazil to eliminate leprosy and provide more solid research approaches to measure the risk or vulnerability in some communities, the aims established were to outline a case profile according to the operational classification of the disease and to identify areas of greater and lesser risk for the occurrence of leprosy.

## Methods

### Study design

A descriptive and ecological study was performed [13].

### Study context

**Fig 1:**
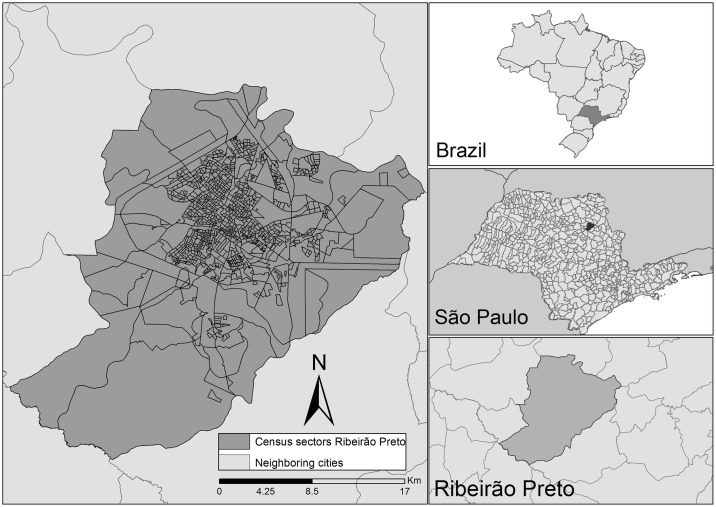
Map showing the location of the city in the state of São Paulo, Brazil (2006–2013).

Ribeirão Preto is a city in the interior of the state of São Paulo (Fig 1), located at 47°48’24”W longitude and 21°10’42”S latitude, 314 Km from the state capital São Paulo and 697 Km from Brasília. The city has an area of approximately 650 Km^2^ and a high demographic density of 995.3 inhabitants per Km^2^. The estimated population in 2010 corresponded to 647,862 inhabitants, 99.7% of whom lived in urban areas^14^.

Concerning the social and economic indicators, the city ranks in Group 2 of the São Paulo Social Responsibility Index (IPRS), that is, with high levels of wealth but unsatisfactory social indicators [15]. The Municipal Human Development Index (IDHM) corresponds to 0.80, the Poverty Index to 11.75% and the Gini Index to 0.45 [14,15].

Concerning the Health Care Network, the city is divided into five Health Districts (DS), North, South, East, West and Central, with a total of 49 Primary Health Care services, including five District Primary Health Care Services (UBDS), 18 Family Health Services (USF) and 26 Primary Health Care Services (UBS), with a total coverage of 22.27% of the population of Ribeirão Preto [16]. The hospital network includes 15 institutions, including one University Hospital, nine hospitals affiliated with the public network of the Brazilian National Health System (SUS) and five non-affiliated hospitals [16]. Regarding leprosy, the entire service is concentrated in three clinics: *Centro de Referência em Especialidade Central*, *Centro de Referência José Roberto Campi* and *CSE Sumarezinho* [17].

### Study population

The study population consisted of cases of leprosy diagnosed by the health services between January 1^st^ 2006 and December 31^st^ 2013.

#### Research variables

The following variables were selected for the study: Date of diagnosis, date of birth, gender, ethnic origin, education, clinical form, operational classification, assessment of grade of physical disability at diagnosis, assessment of physical disability at the moment of cure, number of contacts examined, address, neighborhood, number and zip code, selected according to the information on the Leprosy Reporting/Investigation Forms registered in the Brazilian Disease Notification System (SINAN).

#### Data collection procedure

The cases registered in SINAN living in the city of Ribeirão Preto were surveyed. The data were collected between August 31^st^ and September 04^th^ 2015 from the Epidemiological Surveillance Division of the Ribeirão Preto Municipal Health Department (SMS RP).

### Data analysis

In the phase of exploratory analysis of the data, initially, the descriptive analysis of the data was performed using the Statistica version 12.0 software, calculating central tendency measures for the continuous variables and absolute and relative frequencies for the categorical variables. The continuous variable age was categorized.

To analyze the profile of the leprosy cases according to the clinical forms, the dependent variable (operational classification of PB or MB leprosy) was crossed with the independent variables (age, gender, ethnic origin, education, assessment of physical disability at diagnosis, assessment of physical disability at cure, number of contacts examined), applying the chi-square association test with Yates’ correction or Fisher’s exact test. The probability of type I error was set at 5%.

To detect the risk for spatial and spatial-temporal clusters of the leprosy cases, the cases were geocoded, using the TerraView version 4.2.2 software, standardizing and equalizing the addresses of the resident cases in the urban zone of the city with the StreetBase digital address map in UTM projection—Zone 23S/WGS1984 available in the Shapefile extension, purchased from the *Imagem Soluções de Inteligência Geográfica* company. In this phase, cases with blank or incomplete addresses were ignored, as were cases in rural areas and cases in which the address was the municipal prison.

Geocoding was obtained through the linear interpolation of the full address, including the zipcode, with a point in a corresponding address segment, which permitted patterns of event points to be set up. In addition, for the registers not located in the cartographic database, the *Google Earth* open access tool was used, in which the Geographic Coordinates of the addresses (latitude and longitude) were found.

It should be highlighted that the census sectors were used as the ecological analysis units, with the advantage of being the most disaggregated level of population and socioeconomic groups, collected systematically, periodically and according to a national standard [18]. The cartographic database of the census sectors in Ribeirão Preto was obtained from the website of the Brazilian Institute of Geography and Statistics (IBGE), which consists of 1004 census sectors, of which only 988 were considered due to representing to the urban zone of the city and presenting a resident population [19].

Next, the scanning spatial analysis technique was used, which was developed by Kulldorff and Nagarwalla [20] to detect clusters in space and in space and time. The search for clusters is made by placing a circle with the radius of the variable around each centroid and calculating the number of events within the circle. If the coefficient observed for the region delimited by the circle, called region z, is higher than expected, the circle is called a cluster. This procedure is repeated until all centroids have been tested [21].

Based on this situation, the hypotheses formally elaborated to detect clusters were H_0_: there are no clusters in the region of Ribeirão Preto and H_1_: region z is a cluster. To identify essentially spatial clusters, the SaTScan 9.4 software was used and, as the events studies (leprosy cases) were counts and rare in relation to the population, Poisson’s discrete model was used. Thus, the following conditions were adopted: no geographical overlapping of the clusters, maximum cluster size equal to 50% of the population exposed, circular cluster and 999 replications. It should be highlighted that, in this phase, the information on the year of occurrence of the event was not used.

As well as permitting the spatial analysis, the scanning statistics also permitted the incorporation of the temporal factor, in which the identification of clusters of events [22] simultaneously in space and time is of interest. Thus, the SaTScan 9.4 software was also used to detect spatial-temporal clusters, under the same conditions as defined above for the spatial clusters, however, considering the maximum size of the temporal cluster as equal to 50% of the study period, the precise time, as day, month, year, and the time period between 2006 and 2013.

In addition, the spatial and spatial-temporal scanning techniques were processed, controlling for the occurrence of cases by population size of the census sectors, by their age distribution and according to gender, as well as attempts to detect high and low relative risk (RR). The relative risk allows information from distinct areas to be compared, standardizing it and removing the effect of the different populations, therefore showing how intensely a certain phenomenon occurs in relation to all other study regions [12,13].

A p-value <0.05 was adopted for statistically significant clusters. Clusters that considered only one census sector and presented zero cases of leprosy were ignored. Furthermore, thematic maps were constructed from the scanning analyses, containing the RR of the clusters obtained using the ArcGIS 10.1 software.

### Ethical aspects

Approval for the study was obtained from the Research Ethics Committee of the Ribeirão Preto College of Nursing, with Evaluation No. (CAAE) 44637215.0.0000.5393. Signing of a consent form was not necessary as secondary data were used and the participants were not identified.

## Results

### Case profile according to operational classification of the disease

In total, 434 cases of leprosy were identified, with a predominance of males (n = 264; 60.83%) between 15 and 59 years of age (n = 297; 68.43%). Concerning education, 244 (56.22%) subjects had complete or incomplete elementary education and 10 (2.30%) had not attended school. Regarding ethnicity, 237 (54.61%) subjects referred to themselves as white and 118 (27.19%) mixed race. Considering the clinical form of the disease, it was observed that 188 (43.31%) presented the dimorphic form and 101 (23.28%) the lepromatous form.

In Table 1, analyzing the crossing between the PB or MB operational classification and the independent study variables, a statistically significant association (p = 0.013) was found for gender, with higher proportions of MB cases in males (n = 197; 45.39%).

**Table 1:** Sociodemographic and clinical profile of the leprosy cases according to operational classification. City in the state of São Paulo, Brazil (2006–2013).

Variables	Operational classification				P value
	Paucibacillary (PB)		Multibacillary (MB)		
	n	%	n	%	
**Age (n = 434)**					
<15 years	8	1.84	7	1.61	0.076
15 to 59 years	90	20.74	207	47.70	
>60 years	31	7.14	91	20.97	
**Gender (n = 434)**					
Male	67	15.44	197	45.39	0.013*
Female	62	14.29	108	24.88	
**Ethnicity (n = 403)**					
White	80	19.85	157	38.96	0.127
Black	6	1.49	29	7.20	
Yellow	4	0.99	7	1.74	
Mixed	28	6.95	90	22.33	
Indigenous	1	0.25	1	0.25	
**Education (n = 332)**					
Illiterate	2	0.60	8	2.41	0.841
Elementary education	76	22.89	168	50.60	
High School education	15	4.52	37	11.14	
Higher education	9	2.71	17	5.12	
**Physical disability at diagnosis (n = 377)**					
Grade 0	55	14.59	97	25.73	0.020*
Grade 1	51	13.53	123	32.63	
Grade 2	8	2.12	43	11.41	
**Physical disability at cure (n = 282)**					
Grade 0	71	25.18	96	34.04	0.000*
Grade 1	3	1.06	42	14.89	
Grade 2	0	0.00	28	9.93	
Not assessed	12	4.26	30	10.64	
**Number of investigated contacts (n = 318)**					
Two investigated contacts or less	53	16.67	121	38.05	0.985
More than two investigated contacts	44	13.84	100	31.45	

*p<0.05

The physical disability at diagnosis variable showed a statistically significant association (p = 0.020), with greater proportions of the MB cases with disabilities 1 and 2 (n = 166; 44.04%), when compared to the PB cases.

At the end of the treatment, when the disabilities were assessed, a statistically significant association was again observed (p = 0.000), with greater proportions of disabilities 1 and 2 in the MB cases (n = 70; 24.82%).

#### Spatial and spatial-temporal clusters and risk areas for the occurrence of the disease

Of all cases reported during the study period, 412 cases were standardized for geocoding, with nine cases excluded due to the address being blank and/or incomplete, eight cases due to being in rural areas and five cases due to the Ribeirão Preto penitentiary being indicated as the address. Thus, of the 412 cases, 384 (93.20% cases) were geocoded, using the address database of the city and the TerraView software. A further nine cases were geocoded using Google Earth, totaling 392 geocoded cases (95.14% of the 412 cases).

**Fig 2:**
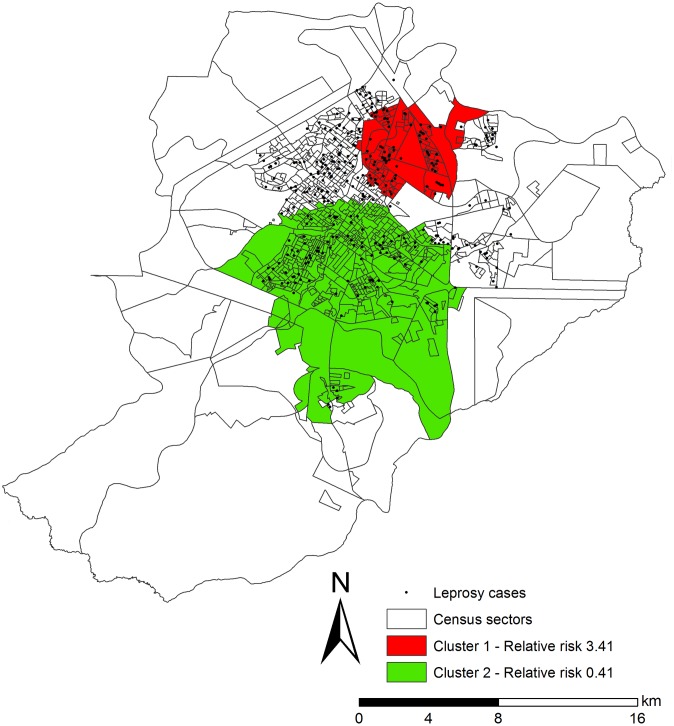
Spatial clusters of leprosy cases, controlled by population of census sectors by gender and age. City in the state of São Paulo, Brazil (2006–2013).

The spatial scanning of the leprosy cases revealed two statistically significant purely spatial clusters (Fig 2).

Spatial cluster 1 (p = 0.000), a high-risk cluster for leprosy, including 90 census sectors, a population of 58,438 inhabitants, 102 cases of leprosy, a mean rate of 22.6 cases per 100,000 inhabitants and a RR of 3.41 (95%CI = 2.721–4.267).

Spatial cluster 2 (p = 0.000), a low-risk (or protection) cluster for the occurrence of leprosy cases, including 477 census sectors, a population of 273,626 inhabitants, 105 cases of leprosy, a mean rate of 4.6 cases per 100,000 inhabitants and a RR of 0.41 (95%CI = 0.512–3.046).

**Fig 3:**
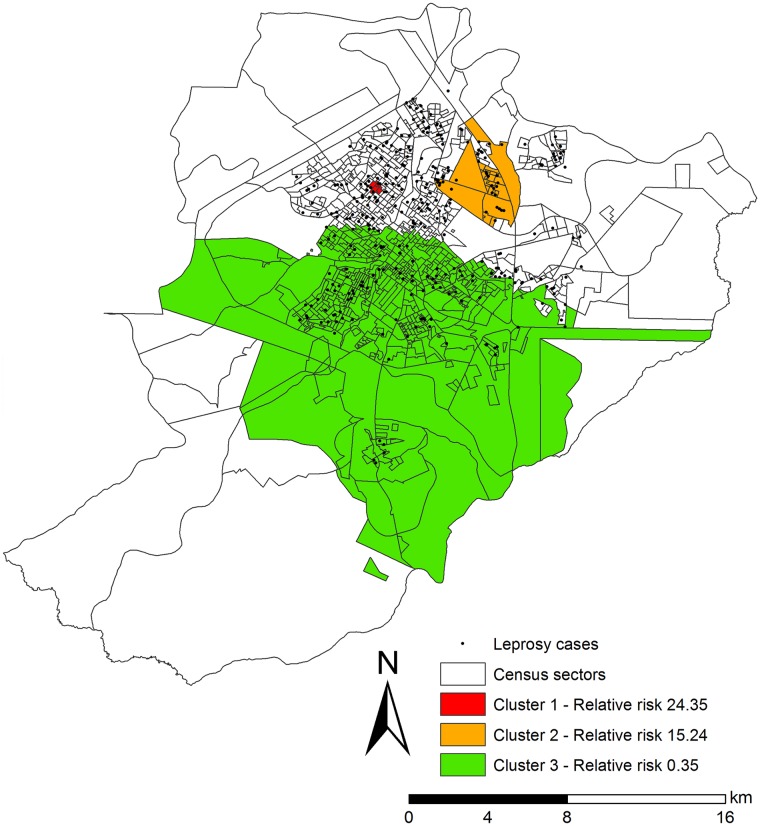
Spatial-temporal clusters of leprosy cases controlled by population of census sectors, by gender and age. City in the state of São Paulo, Brazil (2006–2013).

In the spatial-temporal analysis of the leprosy cases in Ribeirão Preto, three statistically significant clusters were observed (Fig 3).

Cluster 1 (p = 0.006), a high-risk cluster, in the period from 2012 until 2012, covering five census sectors, a population of 3,697 inhabitants, seven leprosy cases, a mean rate of 194.5 cases per 100,000 inhabitants and a RR of 24.35 (95%CI = 11.133–52.984).

Cluster 2 (p = 0.000), a high-risk cluster, between 2012 and 2013, consisting of 25 census sectors, a population of 13,975 inhabitants, 27 cases of leprosy, a mean rate of 115.9 cases per 100,000 inhabitants and a RR of 15.24 (95%CI = 10.114–22.919).

Cluster 3 (p = 0.000), a protection cluster, between 2008 and 2011, consisting of 517 census sectors, a population of 287,899, 41 cases of leprosy, with a mean rate of 3.4 cases per 100,000 inhabitants and a RR of 0.35 (95%CI = 0.488–3.982).

In the strictly spatial analysis, the high-risk cluster was located in the North, West and Central Health Districts of the city. The low-risk cluster identified included areas of the West, East and South Districts.

In the spatial-temporal analysis, the largest cluster was located in the West Health District of the city, and the second largest cluster in the North and West Health Districts. The census sectors with low risk for cases included areas of the West, East and South Districts.

## Discussion

The aim of the study was to outline the case profile according to the operational classification of leprosy and to identify areas of greater and lesser risk for the occurrence of the disease. A statistically significant association was observed for the gender variable, with a higher occurrence of MB forms among men; among the MB forms, more disabilities were also identified at the moments of diagnosis and cure.

Regarding age, it was observed that the subjects most affected by the MB form were between the ages of 15 and 59 years, a phenomenon that has been observed in other contexts in Brazil [23]. This age range refers to the economically active age ranges of the population, in which the health services should concentrate on preventive measures, with the diagnosis, treatment and identification of the appearance and development of lesions, disabilities and reactive conditions. Early intervention avoids or minimizes the high social cost the disease provokes, due to the withdrawal of this population from productive activities.

The significant predominance of male cases in the MB form can be related to the physiopathological mechanisms of the disease. However, this aspect has not been explored nor well clarified in the scientific literature. Other studies have shown that males are more vulnerable, presenting more severe and disabling clinical forms, at the moment of the diagnosis as well as at the time of completing the medication regime [24–26].

Considering females, in the last two decades, there has been a decline in the number of women with the more severe clinical forms, which strengthens the hypothesis raised. However, the literature shows that women tend to visit health services earlier and attend more regularly [27], which should also be considered in the causal line of the disease.

Concerning the educational level of the subjects, despite the lack of a statistically significant association, the highest proportions of MB cases presented elementary education, in accordance with findings of other studies [28]. A case-control study by Kerr-Pontes [28] suggested that low education acts as a risk factor for the transmission of leprosy. In a study conducted in a city in the state of Tocantins—Brazil, it was suggested that low education is associated with the development of physical disabilities [23].

The number of disabilities at the moment of diagnosis and at the end of the treatment among MB cases was also highlighted in the study, as an aspect that should be considered in the planning and organization of the health services.

Studies indicate that MB patients present 5.7 times greater chance of presenting disabilities, mainly at the end of treatment, when compared to PB patients [29]. The result raises the hypothesis that MB cases are diagnosed later, with a higher grade of neural commitment, which can favor the development of disabilities, closely linked to the time factor [30].

The disabilities reflect the late diagnosis and the quality of care delivered in the context of the health services. If the disability staging of the patient evolves, the technologies the health agents use are, most probably, not suitable or in accordance with the needs of the patient.

In Brazil [2], it is recommended that the health services, especially Primary Health Care, assess and determine the grade of disability of the leprosy patient at the moment of diagnosis, at least once per year during the treatment and at the end of treatment, identifying and preventing physical deformities as early as possible.

The grade of physical disability can be attributed not only to late diagnosis, but also to neuropathies, to treatment irregularities, mainly related to the administration of MDT, and to self-care advice, such as the use of eyewashes, moisturizers, maintenance of domestic appliances and clothing [23].

The present study also evidenced the predominance of the dimorphic and lepromatous clinical forms, a phenomenon also demonstrated in other research scenarios [31–35]. The transmissible clinical forms, dimorphic and lepromatous, due to their high bacillary load, can be highly disabling and stigmatizing, being the main reservoir of the disease [2,8]. Hence, the large proportion of new cases of these clinical forms indicates errors in the active detection of cases and in the search for communicants [31,36].

Through the scanning statistics, risk areas can be stratified and census sectors identified that are inclined toward the establishment of statistically significant clusters for leprosy, in space as well as in space-time. This method permits the spatial distribution of cases to be understood, testing whether the pattern observed is random, regularly distributed or clustered. It also permits the existence of possible environmental factors and the extent of the infection risk to be identified [37].

The high-risk clusters, in the spatial as well as the spatial-temporal analysis, included the West, North and Central Health Districts, which comparatively showed great similarities in the socioeconomic profile and in the occupation profile of their populations. The North Health District had the lowest social indicators among the five health districts studied, with the highest percentage of people earning less than one minimum wage, the lowest Gross School Attendance Rate (TBFE), the lowest Municipal Human Development Index in Education (MHDI-E) and the largest number of subnormal clusters (communities) in the city [38].

In the West District, the health network was more complex, having the largest number of SUS health services, with the second highest percentage of exclusive users. A high percentage of residential housing was identified, with a considerable number of residents per household and a predominance of households with incomes between one and five minimum wages. The Central Health District, in turn, consisted of the oldest neighborhoods of the city, with a traditional service network, without coverage of the Family Health Strategy (FHS), with the main referral center for the treatment of leprosy cases situated there [38]. The district presented a significant percentage of people over 60 years of age and a high burden of chronic health conditions among its inhabitants.

Concerning the low-risk areas identified in the study, it was noticed that the East and South Health Districts corresponded to the protection areas in the spatial and spatial-temporal analyses. The East and South Districts were both characterized by good TBFE and MHDI-E rates and a low rate of population without income. It should be highlighted that the East District possessed the highest percentage of families earning 50 or more minimum wages and the lowest percentage of exclusive SUS users.

The low-risk areas in the city should be highlighted and analyzed with caution in terms of the spatial behavior of leprosy, as the representation of these clusters can evidence errors in the detection of causes by the health services in the “protection” areas for leprosy. This can result in underreporting and/or late diagnosis, serving as an alert for the need to intensify active search actions in order to detect a larger number of cases earlier in these regions [39].

The risk clusters estimated by the scanning statistic revealed the focal and unequal behavior of leprosy among the regions of the city, showing that neighborhoods in the North, West and Central Districts presented populations at high risk of catching the disease. The relationship between the disease and these regions can be associated with the fact that they include the neighborhoods with the greatest social inequalities [35,38,40], with precarious housing, many residents per household, low income and low education, which are factors that favor the dissemination of leprosy [37].

In this context, studies demonstrate that populations exposed to social vulnerabilities, such as unhealthy housing conditions, basic sanitation deficits, low per capita income, irregular occupation of places unsuitable for habitation and many people sharing the same house, represent the main factors for leprosy [18,31,34,36,41,42,43], which, in terms of spatial distribution, presents the focal behavior the disease, as evidenced in earlier studies [1,35,44,45,46,47,48].

It should be highlighted that places with many people living in the same house are the main source of maintenance of the leprosy transmission chain and form the domestic contacts. People living near individuals with leprosy and their social contacts have a greater risk of infection [49]. Frequent clusters are not only associated with lower socioeconomic levels and populational clusters, but are also related to shortages in health services [31].

Concerning the constitution of the care network for leprosy patients, the city follows a centralized and verticalized model, in which municipal referral centers serve as the main access for the entry and follow-up of cases. The municipal care flowchart recommends that, when primary health care services identify a suspected case of the disease, they should forward it to the municipal referral centers to confirm the diagnostic hypothesis and monitoring of the case.

Although the current strategies for the control and elimination of the disease have provided positive results, mainly in the last three decades, they are still considered insufficient to eliminate the disease. The condition identified as essential to achieve the target of elimination of the disease, as proposed by the WHO, is the increased supply of health services through the decentralization of control actions in the cities and the inclusion of leprosy treatment in the Primary Health Care services, mainly in areas of social inequality [9].

The strengthening of PHC, with improved access to the health services, the earlier and more effective detection of leprosy cases, the active search for communicants by Community Health Agents’ (ACS) and the free distribution of MDT are essential strategic actions to eliminate the disease [45]. However, other measures attributed to PHC should be highlighted. The community’s understanding of leprosy (its transmission mechanisms, clinical manifestations and treatment) should be widely discussed and valued, in order to empower the population and reduce the stigma the disease causes, which is still one of the main factors that impede the elimination of leprosy [50]. The advance towards the elimination of leprosy should be based on contact surveillance, on the prevention of multidrug resistance, on the prevention and rehabilitation of physical disabilities and on the reduction of the stigma associated with the disease [5].

The results of this study suggest that the distribution of leprosy is restricted to spaces where a number of factors coincide for its production, including environmental, individual, socioeconomic and health service organization factors.

In the last two decades, spatial analysis has been frequently used as a leprosy control tool in Brazil and in countries with a high prevalence of the disease [44,51]. According to the WHO, this is an effective management tool for disease elimination programs, with its use being recommended in all endemic countries [52]. The identification of spatial clusters through an ecological study is a strategic tool to enhance the understanding about the distribution of a disease and a health resource allocation tool [37]. Being a neglected endemic disease and a severe public health problem, knowledge about the spatial distribution of leprosy and identification of risk areas for the disease are fundamental for its control, being important measures to improve the surveillance actions in certain locations [45].

This study only considered the secondary data available, therefore there might have been underreporting of cases in the areas with lower spatial risk.

These findings contribute to the identification of risk areas for leprosy, presenting elements to consider in the organization and strengthening of the health services in these places in terms of the active search for cases. Considering the early diagnosis and the empowerment of this population, awareness about leprosy needs to be increased and people encouraged to work in partnership with the health services to deal with the problem. Furthermore, social institutions within these areas, such as churches, kindergartens and schools, should be mobilized to work jointly with the health services, as they are located in risk areas.

The study permits a reflection about the health services in these clusters and the mechanisms or technologies these services use to provide access for leprosy patients. Due to the number of grade 2 disabilities found, advances are needed, not only in early diagnosis, but also in the management of cases throughout the treatment, preventing patients from becoming worse after the diagnosis. Therefore, another possibility is the introduction of self-care workshops, preparing patients and families to cope with the disease, with the taboos, prejudices and stigma, and demonstrating how to avoid disabling accidents in the home and work environments, permitting their physical and social rehabilitation. In view of the above, the study contributes to the advance of knowledge in this area and to advances towards the eradication of leprosy in Brazil.
